# The Right to Pay for Hospital Treatment

**Published:** 1910-01-22

**Authors:** 


### Hospital Problems which Press.

### THE RIGHT TO PAY FOR HOSPITAL TREATMENT.

### I.—THE PATIENT'S POINT OF VIEW.

In The Hospital of December 25, 1909, pages 356-8, we have shown by figures taken from the audited statements of British hospitals that the voluntary system of hospital support in this country is adequate, and that it produces on the whole a good margin of revenue over expenditure. In view of this fact it has often puzzled us why it is that from time to time letters and papers find their way into the Press declaring the exact contrary, and advocating that all British hospitals shall be placed upon the rates. We knew, of course, as the result of many years' experience, that when a hospital secretary of the weaker type finds himself in a difficulty in regard to funds, especially if he be a relatively young man, he at once puts himself in line with the degenerate modern fashion of asking for money from the State. This fever of relative incapacity and dependence is fortunately seldom met with nowadays, so we must turn to some other cause for the continuance of an advocacy of State support in the lay Press.

Voluntary Hospitals and Paying Patients.

As the result of much inquiry and investigation, we have arrived at what we believe to be the underlying cause of the repeated demand for State support and control of British hospitals of all types. Wherever in other countries the State or the municipality is in the habit of contributing in greater or less proportion to the maintenance of general and other hospitals, there the hospitals provide for the reception of patients who pay in accordance with their means for hospital care and treatment. The voluntary hospitals have, however, failed to appreciate that a very large proportion of patients, whose circumstances compel them when seriously ill to seek admission to a hospital bed, desire very earnestly to have an opportunity of contributing towards the cost to which they may put a voluntary hospital. For 35 years the admission of pay patients to voluntary hospitals, under regulations which will do justice to all the interests concerned, has been advocated by representative hospital managers and some of the most thoughtful and able members of the medical profession. The system took root in connection with the cottage hospitals, which for 50 years have followed this plan to their financial advantage and the satisfaction of their patients. The special voluntary hospitals in larger cities, at any rate, have adopted a modified form of patients' payments in the out-patient department, which has produced an income in some cases so large as to defray the greater portion of the expenditure. Thus the revenue derived by special hospitals from patients' payments in 1907 was £23,500, or nearly 11 per cent. It was upwards of 16 per cent. in the case of provincial special hospitals, and amounted to no less than 21.6 per cent. in the case of Irish special hospitals. No portion of the revenue of the voluntary hospitals in Scotland is derived from payments by in-patients. On the other hand, although some of the general hospitals in London and elsewhere have adopted a modified form of patients' payments in the out-patient department, usually that by payment of a small fee for medicine, etc., the total revenue for all the general hospitals from patients' payments during the same year in London was only 2.6 per cent., in the provinces 2.2 per cent., in Scotch hospitals 2.9 per cent., and in Irish hospitals 8.2 per cent.

Wanted, a Modified System.

The figures we have quoted show that the principle of payments by patients attending voluntary hospitals has been generally accepted, and it seems to us a great pity that steps should not be taken to meet the general wish of the public in this matter by setting up a plan under which in-patients who desire to pay for their treatment as in-patients in a voluntary hospital may be afforded an opportunity of doing so. In other countries the adoption of this principle by State-supported hospitals has proved a success, and has met a public want. The financial position of the voluntary hospitals, as we have shown, was never better than it is to-day. It is consequently a good time for the managers of voluntary hospitals throughout the kingdom to look into this question and to deal with it on its merits. We believe that if this were to be done, and each voluntary hospital was to prepare a budget at the commencement of each year for the ensuing twelve months, and in connection with it to set aside so many beds as free beds, and so many for the reception of pay patients, hospital subscribers and the public would welcome this innovation, with results which would be wholly beneficial to the popularity, efficiency, and revenues of our voluntary hospitals. We are confident that every important voluntary hospital, and for that matter every special hospital too, would be well-advised to modify the present system of admission of in-patients by classifying the beds, so as to enable every class of the community up to those who could afford to pay an inclusive rate equal to the total cost of a patient's maintenance and treatment in the voluntary hospital to obtain admission at varying rates of payment up to the full cost involved. If this system were to be brought into force by every voluntary hospital it would prove an important addition to the revenue, which would tend immeasurably to strengthen the voluntary system of hospital support and be attended by great advantages to all classes of the community. It has been shown over and over again that the idea that it is wrong for a voluntary hospital to admit pay patients will not bear critical examination. Voluntary hospitals exist to meet the requirements of the people of the day and generation which they serve, and as the British people, to their credit, in increasing numbers have a wish to bear as large a portion of the cost of the hospital treatment as their means afford, it is the duty of every well-managed hospital to meet this public requirement, and to meet it adequately.

